# Disentangling Multidimensional Spatio-Temporal Data into Their Common and Aberrant Responses

**DOI:** 10.1371/journal.pone.0121607

**Published:** 2015-04-22

**Authors:** Young Hwan Chang, James Korkola, Dhara N. Amin, Mark M. Moasser, Jose M. Carmena, Joe W. Gray, Claire J. Tomlin

**Affiliations:** 1 Department of Electrical Engineering and Computer Sciences, University of California, Berkeley, CA, USA; 2 Department of Biomedical Engineering and the Center for Spatial Systems Biomedicine, Oregon Health and Science University, Portland, OR, USA; 3 Department of Medicine, Helen Diller Family Comprehensive Cancer Center, University of California, San Francisco, CA, USA; 4 Department of Electrical Engineering and Computer Sciences, Helen Wills Neuroscience Institute, University of California, Berkeley and UCB/UCSF Graduate Program in Bioengineering, CA, USA; 5 Faculty Scientist, Life Sciences Division, Lawrence Berkeley National Laboratory, Berkeley, CA, USA; Swiss Institute of Bioinformatics, SWITZERLAND

## Abstract

With the advent of high-throughput measurement techniques, scientists and engineers are starting to grapple with massive data sets and encountering challenges with how to organize, process and extract information into meaningful structures. Multidimensional spatio-temporal biological data sets such as time series gene expression with various perturbations over different cell lines, or neural spike trains across many experimental trials, have the potential to acquire insight about the dynamic behavior of the system. For this potential to be realized, we need a suitable representation to understand the data. A general question is how to organize the observed data into meaningful structures and how to find an appropriate similarity measure. A natural way of viewing these complex high dimensional data sets is to examine and analyze the large-scale features and then to focus on the interesting details. Since the wide range of experiments and unknown complexity of the underlying system contribute to the heterogeneity of biological data, we develop a new method by proposing an extension of Robust Principal Component Analysis (RPCA), which models common variations across multiple experiments as the lowrank component and anomalies across these experiments as the sparse component. We show that the proposed method is able to find distinct subtypes and classify data sets in a robust way without any prior knowledge by separating these common responses and abnormal responses. Thus, the proposed method provides us a new representation of these data sets which has the potential to help users acquire new insight from data.

## Introduction

**Fig 1:**
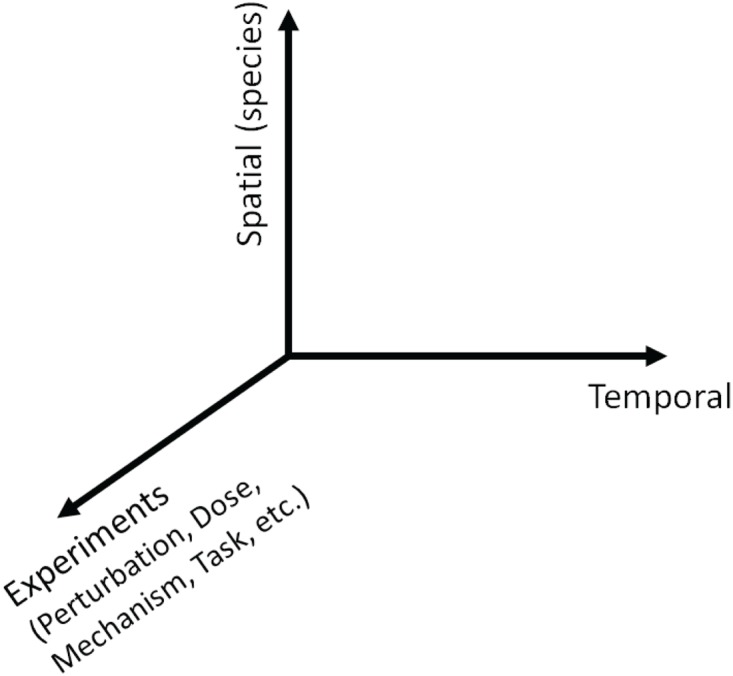
Multi-dimensional spatio-temporal data. We consider various experiments with different perturbations, doses, mechanism, tasks, etc.

Over the last years, the use of high-throughput measurement data has become one of the most exciting trends and important themes in science and engineering. This is becoming increasingly important in biology. However, handling and analyzing biological data have challenges all of their own because these data sets are typically heterogeneous, stemming from a wide range of experiments (Fig 1) and representing the (unknown) complexity of the underlying system [1]. For instance, in molecular biology one may think of the experiment axis in Fig 1 as experimental parameters and conditions, such as cell type, chemical perturbation and genetic alteration. Also, in cancer cells, more specifically the breast cancer that we study [2], since pathway-targeted therapies lead to abnormal behaviors and different responses to external stimuli, challenges occur in analyzing inherently heterogeneous data.

With the growth of the amounts of various biological data, a general question is how to organize the observed data into meaningful structures and how to find an appropriate similarity (or dissimilarity) measure which is critical to the analysis. Since such multidimensional spatio-temporal (note that we refer to “spatio-” as “different species” such as different proteins or different neurons in this paper) data have the potential to provide new insight across multiple dimensions, these data can enable users to start to develop models and draw hypotheses that not only describe the dynamic interactions between states such as genes or neurons but also inform them about commonalities and differences across experimental conditions. A significant challenge for creating suitable representations is to continue handling large data sets and to effectively deal with the growing diversity and quantity of the data sets.

A natural way of viewing these complex high dimensional data sets is to examine and analyze the large-scale features and then to focus on the interesting details. The decomposition enables focusing on the precise effects of each particular feature by placing emphasis on the commonalities or the unique behaviors. For example, the potential of clustering to reveal biologically meaningful patterns in microarray data was first realized and demonstrated in an early paper by Eisen *et al* [3]. Thereafter, in many biological applications, different methods have been used to analyze gene expression data and characterize gene functional behavior. Among various data-driven modeling approaches in biological systems, clustering methods are widely used on various biological data to categorize them with similar expression profiles. However, until recently, most studies have focused on the spatial, rather than temporal, structure of data. For instance, neural models are usually concerned with processing static spatial patterns of intensities without regard to temporal information [4]. Since many existing data-driven modeling approaches such as clustering or classification using biological data focus on static data, they have limitations in analyzing multi-dimensional spatio-temporal data sets.

Recently, much research has focused on time series high-throughput data sets. These data sets have the advantage of being able to identify dynamic relationships between genes or neurons since the spatio-temporal pattern results from the integration of regulatory signals through the gene regulatory network or electrochemical signals through the neural network over time. For example, time series gene expression data sets with various drug-induced perturbations provide the distinct possibility of observing the cellular mechanisms in action [5]. These data sets help us to unravel the mechanistic drivers characterizing cellular response and to break down the genome into sets of genes involved in the related processes [6]. Also, several recent studies focus on the temporal complexity and heterogeneity of single-neuron activity in the premotor and motor cortices [4] [7] [8]. Therefore, instead of concentrating on steady state response, monitoring dynamic patterns provides a profoundly different type of information. Moreover, since many current and emerging cancer treatments are designed to inhibit or stimulate a specific node (or gene) in the networks and alter signaling cascades, advancing our understanding of how the system dynamics of these networks is deregulated across cancer cells and finding subgroups of genes and conditions will ultimately lead to the more effective treatment strategies [2].

In this paper, we propose a Robust Principal Component Analysis (RPCA)-based method for analyzing spatio-temporal biological data sets over various experimental parameters and conditions. Since we consider multidimensional spatio-temporal biological data sets, we note this goes beyond the results in either clustering steady state gene expression data across various experimental conditions or analyzing the dynamic behavior of the system for a particular experimental condition. To demonstrate that our method helps users acquire insight efficiently and to emphasize that the proposed method can be applicable to various domains, we consider two different systems 1) neural population dynamics and 2) a gene regulatory network. The proposed method is intended to aid analysis of dynamic behavior of the system under various experimental parameters or conditions, by retrieving common dynamical information and focusing on the interesting details with a new perspective on the problem. The ultimate goal is to use such information to learn more about the system by acquiring new insight from data.

## Background

### 2.1 Overview: Neural Population Dynamics and Gene Regulatory Network

#### 2.1.1 Neural Population Dynamics

Neural ensemble activity is typically studied by averaging noisy spike trains across multiple experimental trials to obtain an approximate neural firing rate that varies smoothly over time. However, if neural activity is more a reflection of internal neural dynamics rather than response to external stimulus, the time series of neural activity may differ even when the subject is performing nominally identical tasks [8]. In [7], Churchland *et al*. showed that neural activity patterns in the primary motor cortex and dorsal premotor cortex of the macaque brain associated with nearly identical velocity profiles can be very different. This is particularly true of behavioral tasks involving perception, decision making, attention, or motor planning. In these settings, it is critical not to average the neural data across trials, but to analyze it on a trial-by-trial basis [4]. Moreover, stimulus representations in some sensory systems are characterized by the precise spike timing of a small number of neurons [9] [10] [11], suggesting that the details of operations in the brain are embedded not only in the overall neural spike rate, but also in the timings of spikes.

**Fig 2:**
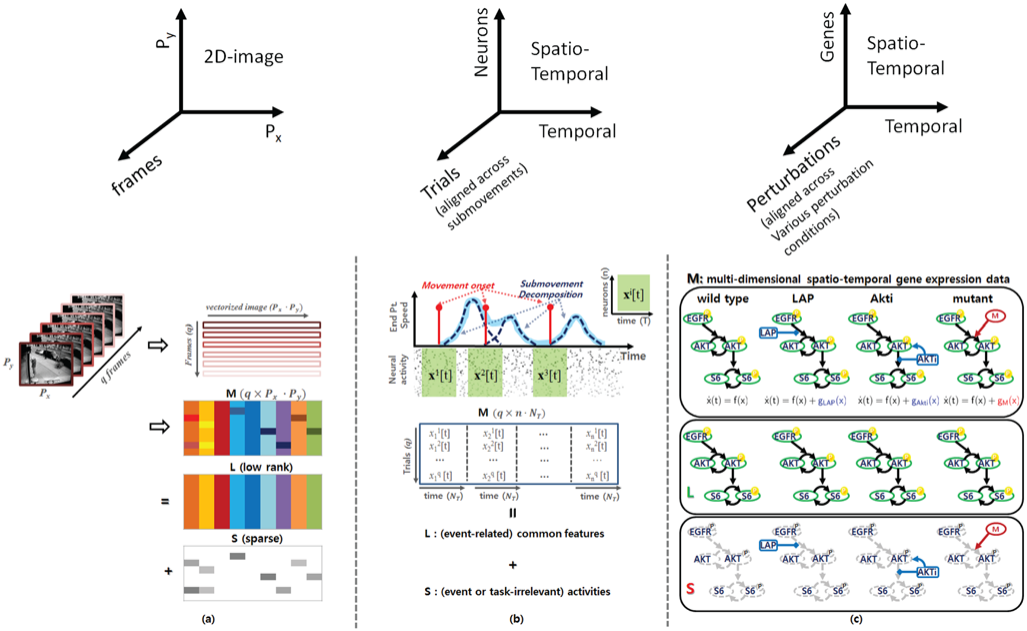
Conceptual representation. (a) RPCA applied to computer vision. A typical example of video surveillance where the low-rank component represents the unchanging background and the sparse component represents the movements in the foreground. (b) RPCA applied to neural systems. The low-rank component putatively represents (submovement relevant) neural signatures and the sparse component represents neural activity unrelated to submovement onset. (c) Collections of drug-induced perturbation experiments and mutant-specific part representations (breast cancer signaling pathway) with **wild-type**, **Lapatinib** treatment, **AKT** inhibitor and **mutant** cell lines where solid black edges represent common network topology, and blue and red edges represent a single change of the network topology for perturbations or mutant cell lines.

The motor and premotor cortices have been extensively studied but their dynamic response properties are poorly understood [4]. Moreover, the role of motor cortex in arm movement control is still unclear, with experimental evidence supporting both low-level muscle control as well as high-level kinematic parameters. We can define the motor cortical activity, which represents movement parameters as per eq (1), and the dynamical system that generates movements as per eq (2) [4]:

$$\begin{array}{ccc} x_{i}\left(t\right) & = & h_{i}\left({\text{param}}_{1}\;(t),{\text{param}}_{2}\;(t),{\text{param}}_{3}\;(t),\ldots \right) \end{array}$$ (1)

$$\begin{array}{ccc} \dot{x}\left(t\right) & = & f(x(t))+u(t) \end{array}$$ (2)

where *x*_*i*_(*t*) is the firing rate of neuron *i* at time *t*, *h*_*i*_ is its tuning function, and each param_*j*_ may represent a movement parameter such as hand velocity, target position or direction. In (2), $x\in R^{n}$ is a vector describing the firing rate of all neurons where *n* is the number of neurons, $\dot{x}$ is its derivative, *f* is an unknown function, and **u** is an external input. In (2), neural activity is governed by the underlying dynamics *f*(⋅), so the characteristics of dynamical system should be present in the population activity. Since we will align spatio-temporal neural activity with the same temporal condition as shown in Fig 2(b), we may be able to extract these characteristics.

#### 2.1.2 Gene Regulatory Network

In microarray data, missing and corrupted data are quite common and not uniform across samples, which include arbitrary corruptions by measurement noise, improper use of biomarker or human error during biological experiments. Two strategies for dealing with missing values are either to modify clustering methods so that they can deal with missing values, or impute a “complete” data set before clustering [12].

Consider collections of time series gene expression of breast cancer cell lines or microarray data sets from pathway-targeted therapies involving drug-induced perturbation experiments. When a specific gene is perturbed as shown in Fig 2(c), the broad gene expression levels of other genes might be perturbed over time. Thus, comparing gene expression levels in the perturbed system with those in the unperturbed system reveals the extra information that is the different cellular mechanisms in action. A dynamical system of the gene regulatory network can be modelled as follows:

$$\begin{array}{c} \dot{x}\left(t\right)=\{\begin{array}{cc} f(x(t))\;\;\;\;\;\;\;\;\;\;\;\;\;\;\text{(without}\;\text{perturbation}\;\text{or}\;\text{wild-type)}\;\;\; & \\ f\left(x\left(t\right)\right)+g_{\left\{\cdot \right\}}\left(x\left(t\right)\right)\;\text{(perturbed}\;\text{or}\;\text{mutant-specific}\;\text{part)} \end{array} \end{array}$$ (3)

where $x(t)\in R^{n}$ denotes the concentrations of the rate-limiting species, $\dot{x}(t)$ represents the change in concentration of the species over time *t*, *n* is the number of species, *f*(⋅) represents the vector field of the typical dynamical system (or wild-type) and *g*_{⋅}_(⋅) represents an additional perturbation or mutant-specific vector field (blue and red edges in Fig 2(c)). For example, small molecule inhibitors such as **Lapatinib** and **AKT** inhibitor can be modeled as additional vector fields such as *g*_LAP_(**x**(*t*)); *g*_AKTI_(**x**(*t*)) which are assumed to be sparse because small molecule inhibitors only affect a single gene expression. Also, even some mutations such as kinase domain mutation, we can simply add a single vector field such as *g*_M_(**x**(*t*)). In other words, we have a unified model for wild-type cell line, $\dot{x}(t)=f(\text{x}(t))$ and in the mutant or perturbation case, we invoke a single change to the network topology or add a single influence for a specific gene (*g*_{⋅}_(⋅)). Here, additional vector fields such as *g*_LAP_(⋅), *g*_AKTI_(⋅) and *g*_M_(⋅) are assumed to be sparse (i.e., affect only a single gene expression). Although these additional vector fields affect only a single gene expression at time *t*, their influence can be propagated through the network over time.

### 2.2 Motivation

Extracting meaningful dynamic features from a heterogenous data set such as spatio-temporal neural activities or time series gene expression data with different perturbations is often intractable for methods sensitive to outliers or noise. In this paper, we consider the task of retrieving such common dynamic features under the presence of inherent outliers, incorporating for example, task-irrelevant neural activities or aberrant responses of gene expression caused by drug-induced perturbation.

The key idea is that despite the inherent heterogeneity of these data, these common dynamics may lie on a lower dimension as compared to the overall heterogeneous dynamics. For example, although gene regulatory network may respond differently to drug-induced treatments, these dynamics still share a fair part of their dynamics and thus the common dynamic behavior should be present in their dynamic responses. Similarly, for spatio-temporal neural activities, some portion of the variability may reflect key features in neural activities corresponding to a specific task even though the responses of each neuron may be corrupted by task-irrelevant neural responses which may vary significantly across many trials. By understanding the shared dynamic properties across different experiments, we can extract the common responses and by isolating the common dynamic behavior, the aberrant responses show how the gene regulatory network operates differently or represent task-irrelevant neural responses. Note that we do not need any *a priori* information about the underlying system. Our method is inspired by advances in computer vision, which we briefly discuss in the following section.

### 2.3 Robust Principal Component Analysis (RPCA)

In the computer vision literature [13], an interesting separation problem is introduced where the observed data matrix can be decomposed into an unseen low-rank component and an unseen sparse component. The method called Robust Principal Component Analysis (RPCA) is a provably correct and efficient algorithm for the recovery of low-dimensional linear structure from non-ideal observations, incorporating for example, occlusions, malicious tampering, and sensor failures.

In video surveillance, we need to identify activities that stand out from the background given a sequence of video frames [13]. Fig 2(a) shows that if we stack the video frames as rows of a matrix $M\in R^{q\times P_{x}\cdot P_{y}}$ where *q* is the number of frames for a given time window, and *P*_*x*_ and *P*_*y*_ represent the number of pixels of 2-D images respectively, then across each row of **M**, there exists a common component that is the stationary background and a changing component which is the moving object in the foreground at each image frame. Here, the data matrix **M** is an input for RPCA and the output is both the stationary background represented as a matrix $L\in R^{q\times P_{x}\cdot P_{y}}$ and the moving objects in the foreground represented as a matrix $S\in R^{q\times P_{x}\cdot P_{y}}$. Intuitively, with only one video frame (i.e., a single static image), the moving objects cannot be identified from the stationary background. However, by stacking all the vectorized frames such that all the frames align across the column direction as shown in Fig 2(a), we can identify the stationary backgrounds which are common variations, and then capture the moving objects which are sparse components for each frame.

With this notion, suppose we are given a large data matrix **M**, which has principal components in the low-rank component and may contain some anomalies in the sparse component. Mathematically, it is natural to model the common variations as approximately the low-rank component **L**, and the anomaly as the sparse component **S**. In [13], Candès *et al*. formulate this as follows:

$$\begin{array}{c} \min_{L,S}{∥L∥}_{*}+\lambda {∥S∥}_{1}\;\text{s.t.}\;M=L+S \end{array}$$ (4)

where ‖**L**‖_*_ denotes the so-called nuclear norm of the matrix **L**, which is the sum of the singular value of **L**, and ‖**S**‖_1_ = ∑_*ij*_∣**S**_*ij*_∣ represents *l*_1_-norm of **S**. A tuning parameter *λ* may be varied to put more importance on the rank of **L** or the sparseness of **S**. Since choosing the tuning parameter *λ* to be $\lambda =1/\sqrt{\max(q,P_{x}\cdot P_{y})}$, works well in practice [13], in the computational results we will present here, we choose the parameter *λ* based on this criteria. However, for practical problems, it is often possible to improve performance by choosing *λ* according to prior knowledge about the solution. Thus, we can also use *λ* as a tuning parameter to trade off more importance between **L** and **S**.

### 2.4 Key Contributions

In [14], Liu *et al*. proposed an RPCA-based method of discovering differentially expressed genes using steady state response. Since they use the static data with different perturbation signals, they only treat the differentially and non-differentially expressed genes for gene identification and thus focus on the spatial structure of data. However, since we focus on the spatio-temporal gene expression data sets with various perturbations, we include the temporal axis as shown in Fig 1. Instead of concentrating on the steady state response [14], analyzing time series gene expression data sets is more relevant to understanding biological systems since it has the distinct possibility of identifying dynamic relationships. With only one time point (i.e., steady state), RPCA may be able to identify outliers or differentially expressed genes at the steady state but it is very limited in its ability to identify drug-specific responses or aberrant responses. By including dynamics, we consider the disentanglement of low-rank and sparse component which results in not only extracting common dynamic features but also detecting specific responses or heterogeneity. As an example, for a gene expression time series data set, when a target protein is perturbed by a specific drug, there are immediate effects on the target protein and compensatory responses on other proteins over time. We can reveal the extra information by comparing protein levels in the perturbed system with those in the unperturbed system. Since abnormal behaviors or different responses to external stimuli or different cell lines can be extracted from the original data using the information available in the data set, we could classify data and reveal biological meaningful patterns, for example, observing distinct cellular mechanisms in action.

Since we treat the spatio-temporal gene expression data set and focus on the relationship between gene regulatory network and dynamics of each regulatory signal, we note this goes beyond the results in [14] [15]. In order to handle multidimensional spatio-temporal responses properly, we propose the strategy for arranging the input data matrix and incorporate with Random Projection (RP) for the preprocessing step. In the following section, we will show why this preprocessing step is necessary for this analysis and present that by using RP, we can handle either a sparse data set (i.e., neural activity) or data sets with eccentric distribution (i.e., proteomic data), which are common in biological data sets. Through numerical and biological examples, we will demonstrate that we can improve the identifiability of the common dynamic features by using RP. Also, we will demonstrate that the proposed method provides us a new representation of biological data which has the potential to acquire new insight from data.

## Methods

### 3.1 How to Construct the Data Matrix **M**

In the video surveillance example shown in Fig 2(a), each row of **M** represents the vectorized 2-D images at each time frame. Since each image consists of the stationary background (**L**_*i*,:_) and the moving objects in the foreground (**S**_*i*,:_) at each time *i*, we denote **M** as follows:

$$\begin{array}{c} M=[\begin{array}{c} M_{1,:}\\ M_{2,:}\\ \ldots \\ M_{q,:} \end{array}]=[\begin{array}{c} L_{1,:}\\ L_{2,:}\\ \ldots \\ L_{q,:} \end{array}]+[\begin{array}{c} S_{1,:}\\ S_{2,:}\\ \ldots \\ S_{q,:} \end{array}]=L+S \end{array}$$ (5)

where **M**_*i*,:_, **L**_*i*,:_ and **S**_*i*,:_ represent the *i*-th row of **M**, **L** and **S** respectively. If there were no moving object in the foreground and no variation for a given video sequence (i.e., ∀*i*, **S**_*i*,:_ = **0**), **L**_*i*,:_ (= **L**_*j*,:_ (*i* ≠ *j*)) would represent the common stationary background. On the other hand, if not (i.e., **S**_*i*,:_ ≠ **0**), **M** represents the aligned corrupted measurements **M**_*i*,:_. Although the measurements are corrupted by moving objects in the foreground, we are able to separate **L** and **S** under certain conditions [13].

#### 3.1.1 Neural Population Dynamics

Recall eq (2) and consider an experiment involving a non-human primate subject instructed to make visually-guided planar reaches with its hand. During the experiment, hand position and velocity, as well as the discharge of neurons from primary motor cortex and dorsal premotor cortex were recorded. See reference [15] for details on the data sets. All procedures were conducted in compliance with the National Institute of Health Guide for Care and Use of Laboratory Animals and were approved by the University of California, Berkeley Institutional Animal Care and Use Committee. Then, hand velocity data were decomposed into a sum of minimum-jerk basis functions where a submovement representation is a type of motor primitive; for example, the hand speed profile as a function of time resulting from arm movements can be represented by a sum of bell-shaped functions as shown in Fig 2(b), each of which is called a submovement [15] and denoted as different trials. In Fig 2(b), each red bar denotes submovement onset, i.e., when the subject triggers submovement.

Suppose we align the spatio-temporal neural activity ${\text{x}}^{i}[t]≜\left[\begin{array}{c} {\text{x}}^{i}(t_{0}),{\text{x}}^{i}(t_{1}),\ldots ,{\text{x}}^{i}(t_{N_{T}-1}) \end{array}\right]\in R^{n\times N_{T}}$ governed by (2) with submovement onset where the superscript *i* represents the *i*-th trial and *N*_*T*_ represents the number of time points for the chosen time window. Then, **M** may be represented as follows:

$$\begin{array}{c} M=[\begin{array}{cccc} x_{1}^{1}\left[t\right] & x_{2}^{1}\left[t\right] & \ldots & x_{n}^{1}\left[t\right]\\ x_{1}^{2}\left[t\right] & x_{2}^{2}\left[t\right] & \ldots & x_{n}^{2}\left[t\right]\\ \ldots & \ldots & \ldots & \ldots \\ x_{1}^{q}\left[t\right] & x_{2}^{q}\left[t\right] & \ldots & x_{n}^{q}\left[t\right] \end{array}]=[\begin{array}{cccc} {𝒳}_{1} & {𝒳}_{2} & \ldots & {𝒳}_{n} \end{array}]≜X\in R^{q\times n\cdot N_{T}} \end{array}$$ (6)

where ${𝒳}_{i}≜\left[\begin{array}{c} {\text{e}}_{i}^{⊤}{\text{x}}^{1}[t];{\text{e}}_{i}^{⊤}{\text{x}}^{2}[t];\ldots ;{\text{e}}_{i}^{⊤}{\text{x}}^{q}[t] \end{array}\right]\in R^{q\times N_{T}}$ represents the temporal neural activity of the *i*-th neuron, $e_{i}\in R^{n}$ is a unit vector, and *q* is the number of trials or submovements. Thus, each row of X represents the vectorized spatio-temporal neural response for the each trial. Note that we align each spatio-temporal data set **x** ^*j*^[*t*] with the same temporal condition (submovement onset) as shown in Fig 2(b) but we do not separate different types of submovement. For example, submovements with different reach directions, or with different ordinal positions in an overlapped series of submovements, are combined in our input matrix X. With the similar notion of the stationary background in video surveillance, some portion of the variability may reflect common dynamic features (**L**) corresponding to triggering submovement even though the responses of each neuron are corrupted by task-irrelevant neural responses (**S**) and may vary significantly across many trials.

#### 3.1.2 Gene Regulatory Network

Recall eq (3) and consider Fig 2(c). In (3), the vector field (*g*_{⋅}_) represents a single influence for a specific gene, yet this single influence can be propagated through the network over time. For example, when we inhibit *x*_*j*_, the *j*-th gene in **x**, the gene expression levels of other genes can be affected indirectly; if *x*_*j*_ is connected with only few genes, this perturbation may only affect a small fraction of the total number of gene expression levels.

Similar to eq (6), we construct X using gene expression time series data with *q* different perturbations and/or different cell lines. Here, each row of $X\in R^{q\times n\cdot N_{T}}$ represents the vectorized time series gene expression $x^{i}[t]\in R^{n\times N_{T}}$ (*n*: the number of genes, *N*_*T*_: the number of time points and *q*: the number of different perturbation conditions including the number of different cell lines) and different rows represent spatio-temporal responses of different perturbations or different cell lines.

Since time series gene expression results from integration of regulatory signals constrained by the gene regulatory network, the input matrix X may reflect common dynamic response corresponding to the characteristics of the network structure. Intuitively, in video surveillance, if someone stays motionlessly in all the frames, the RPCA algorithm discriminates him as a low rank component. Unless he moves, we could not see the background because he always blocks the background. Similarly, in order to extract common response of gene regulatory network exactly, we should perturb the entire network arbitrarily and uniformly.

### 3.2 Random Projection (RP) and Identifiability

In [13], Candès *et al*. discuss the identifiability issue. To make the problem (4) meaningful, the low-rank component **L** must not be sparse. Another identifiability issue arises if the sparse matrix **S** has low-rank. In many computer vision applications, practical low-rank and sparse separation gives visually appealing solutions.

**Fig 3:**
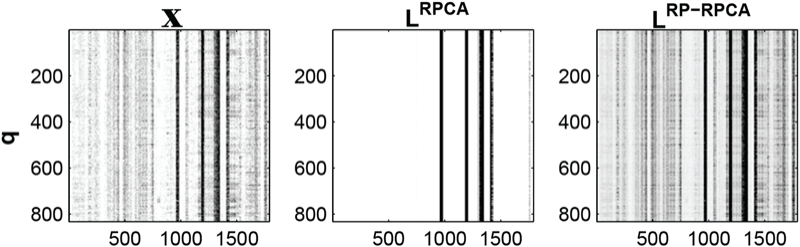
The low-rank matrices from both RPCA and RP-RPCA. 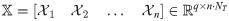
 is an input matrix and we choose *m* = *n* = 64 for the comparison (contrast represents activity of neuron. i.e., high contrast represents highly modulated neural activity and white color represents zero neural activity). (left) raw-data (center) low-rank component using RPCA and (right) low-rank component using RP-RPCA.

However, for neural activity data, only a small subset of the whole ensemble of neurons is active at any moment as shown in Fig 3(left). Since the input matrix X is sparse, the low-rank component **L** might be sparse or the sparse matrix **S** might have low-rank. In addition, the original distributions of the amplitude of individual neuronal activities or gene expressions are highly skewed. For example, neural activities often form very eccentric clusters shown in Fig 3(left); some neurons are highly activated (30-40 spikes/sec) but others typically have only a few spikes per second. Similarly, gene expressions form very eccentric clusters since each gene expression shows different scales in practice. Also, for the pathway targeted therapies, since gene regulatory networks are known to be sparse, a large subset of the whole ensemble of genes might be deactivated at any moment and thus X may be sparse.

These imply that practical low-rank and sparse separation seems to be ambiguous and might present a challenge to achieve biologically meaningful solutions in both neural activity data sets and drug-induced perturbation experiment data sets. To remedy this identifiability issue, we propose the RPCA-based method in conjunction with RP; RP can not only de-sparsify the input data set but also make a highly eccentric distribution more spherical, thus making the singular vectors of the low-rank matrix reasonably distributed. Thus, RP is able to make the input data amenable to this analysis. Moreover, for the gene regulatory network, we can design experiments by perturbing each gene uniformly well.

#### 3.2.1 Random Projection(RP)

Recent theoretical work has identified RP as a promising dimensionality reduction technique. In [16], Dasgupta showed that even if the original distribution of data samples is highly skewed (having an ellipsoidal contour of high eccentricity), its projected counterparts will be more spherical. Since it is conceptually much easier to design algorithms for spherical clusters than ellipsoidal ones, this feature of random projection can simplify the separation into the low-rank and sparse components, and thus we can reduce the computational complexity of the non-smooth convex optimization, in particular *l*_1_ and nuclear norms minimization, used in (4).

By incorporating RP, many speedup methods were developed in optimization by avoiding large-scale Singular Vector Decomposition (SVD). For example, in [17], Mu *et al*. demonstrated the power of the projected matrix nuclear norm by reformulating RPCA and in [18], Zhou *et al*. presented the effectiveness and the efficiency of Bilateral Random Projections. However, both methods [17] [18] consider a dense matrix X and use projection only for reducing computational effort, while in this paper we consider the case in which the input matrix X is not applicable to the problem (4) directly due to sparsity or eccentric distribution in X. In other words, we are not interested in computational efficiency here, but focus on the issues in the input matrix X in order to make the problem (4) meaningful. Otherwise, the result of RPCA may provide the mis-identified result since the input is improper for the problem (4).

As we mentioned earlier, the neural activity data in Fig 3(left) are sparse and for the proteomic data, if the negative perturbation has an effect on down regulation of signaling at the immediate target and other proteins, the corresponding spatio-temporal data set can be sparse. Or, the proteomic data often shows different scales in the measurement across different proteins (i.e., eccentric distribution). Thus, the original input data are not applicable to RPCA analysis directly due to the nature of the input data. For example, with eccentric distribution of the scales in biological data, the low-rank component **L** may be biased since the optimization problem (4) may focus on large scale components in X. Also, if the input data is sparse, the problem (4) cannot be meaningful due to the identifiability issue [13]. Therefore, we use RP for preprocessing step in order to handle this issue properly, and make the input data amenable for RPCA analysis.

The idea of RP is that a small number of random linear projections can preserve key information. Projecting the data onto a random lower-dimensional subspace preserves the similarity of different data vectors, for example, the distances between the points are approximately preserved. Theoretical work [16] [19] [20] [21] guarantees that with high probability, all pairwise Euclidean and geodesic distances between points on a low-dimensional manifold are well-preserved under the mapping $\Psi :R^{n}\to R^{m},m\leq n$. Also, RP can reduce the dimension of data while keeping clusters of data points well-separated [16]. Consider a linear signal model

$$\begin{array}{c} y\left(t\right)=\Psi x\left(t\right)=\sum_{i=1}^{n}x_{i}\left(t\right)\psi_{i}\in R^{m} \end{array}$$ (7)

where **Ψ** = [*ψ*_1_ *ψ*_2_ … *ψ*_*n*_] is an *m* × *n* projection matrix whose elements are drawn randomly from independent identical distributions. First, note that the dimensionality of the data **x** is reduced since *m* ≤ *n*. Also, if we define ${𝒴}_{i}≜\left[\begin{array}{c} {\bar{\text{e}}}_{i}^{⊤}{\text{y}}^{1}[t];{\bar{\text{e}}}_{i}^{⊤}{\text{y}}^{2}[t];\ldots ;{\bar{\text{e}}}_{i}^{⊤}{\text{y}}^{q}[t] \end{array}\right]\in R^{q\times N_{T}}$ where ${\bar{\text{e}}}_{i}$ is *m*-dimensional unit vector and $Y≜\left[\begin{array}{cccc} Y_{1} & Y_{2} & \ldots & Y_{m} \end{array}\right]$, then $Y^{⊤}=(\Psi ⊗I_{N_{T}})X^{⊤}$ or $Y=X(\Psi^{⊤}⊗I_{N_{T}})$ where ⊗ represents the Kronecker product: if **A** is an *m* × *n* matrix and **B** is a *p* × *q* matrix, then the Kronecker product **A** ⊗ **B** is the *mp* × *nq* block matrix

$$\begin{array}{c} A⊗B=[\begin{array}{ccc} a_{11}B & \ldots & a_{1n}B\\ \vdots & \ddots & \vdots \\ a_{m1}B & \ldots & a_{mn}B \end{array}] \end{array}$$

and $I_{N_{T}}\in R^{N_{T}\times N_{T}}$ is an identity matrix. Intuitively, Y represents the mixture of ${𝒳}_{i}$ across spatial directions (i.e., different proteins or neurons) with projection matrix **Ψ** in order to make the singular vectors of the low-rank matrix reasonably distributed. Note that since we are interested in extracting the common dynamic behavior, we keep the temporal order of each experimental data set by the Kronecker product and **I**_*N*_*T*__ (i.e., ⊗ **I**_*N*_*T*__). Thus, RP is only used for transforming data in the space domain.

#### 3.2.2 Identifiability

Suppose our input X in eq (6) can be decomposed as $X=\text{L}+\text{S}=\sum_{i=1}^{d_{L}}\sigma_{i}{\text{u}}_{i}{\text{v}}_{i}^{*}+\sum_{i=1}^{d_{S}}\lambda_{i}{\text{a}}_{i}{\text{b}}_{i}^{*}$ where *σ*_*i*_ are the positive singular values, $u_{i}\in R^{q\times 1}$, $v_{i}^{*}\in R^{1\times n\cdot N_{T}}$ are the left- and right-singular vectors of **L**, and *d*_*L*_ represents the rank of the matrix **L**. *d*_*S*_ is the number of sparse components in **S**, and $a_{i}\in R^{q\times 1},b_{i}\in R^{1\times n\cdot N_{T}}$ are sparse with only one nonzero entry respectively. By using RP, we have for Y,

$$\begin{array}{ccc} Y & = & X(\Psi^{⊤}⊗I_{N_{T}})≜XR=\mathrm{LR}+\mathrm{SR}\\ & = & \sum_{i=1}^{d_{L}}\sigma_{i}u_{i}{\left(R^{⊤}v_{i}\right)}^{*}+\sum_{i=1}^{d_{S}}\lambda_{i}a_{i}{\left(R^{⊤}b_{i}\right)}^{*}\\ & = & \sum_{i=1}^{d_{L}}\sigma_{i}u_{i}{\tilde{v}}_{i}^{*}+\sum_{i=1}^{d_{S}}\lambda_{i}a_{i}{\tilde{b}}_{i}^{*} \end{array}$$ (8)

where we denote (**Ψ** ^⊤^ ⊗ **I**_*N*_*T*__) by **R**. As we mentioned above, our input X is sparse or has eccentric distribution, so the singular vectors of the low-rank matrix **L** might not be reasonably spread out. However, by using RP (multiplying by **R**), the singular vectors ${\tilde{\text{v}}}_{i}$ of the resulting matrix become reasonably spread out.

## Results

### 4.1 Numerical Example

To illustrate the issue of identifiability and how RP can alleviate this issue, we consider a simple example: we generate a sparse low-rank input matrix $X\in R^{50\times 2\cdot 10}$ (*q* = 50, *n* = 2, *N*_*T*_ = 10) where the rank of X is 6 as shown in S1 Fig. (a). Note that in this example we choose the same dimension for the input X and X (refer to (7) and (8), no dimension reduction). This is done so that $\Psi \in R^{m\times n}$ in eq (7) is invertible (we choose *m* = *n* and a nonsingular matrix **Ψ**), allowing us to compare the outputs of RPCA and RP-RPCA directly, which will be described below. Here, by using RP, we take advantage of de-sparsifying our input data and reducing the eccentric distribution. In general, choosing *m* < *n* makes Y much denser because information is compressed by RP.

To evaluate the performance of separation into a low-rank and a sparse component, we add sparse corruption for $X\$:\$X_{corruption}=X+S_{corruption}$ and $Y_{corruption}=X_{corruption}R=XR+S_{corruption}R$ where **R** = (**Ψ** ^⊤^ ⊗ **I**_*N*_*T*__) is the projection so $Y_{corruption}$ is the projected corrupted input $X_{corruption}$. To compare the performance of RP-RPCA with RPCA, we first decompose $Y_{corruption}$ into its low-rank and sparse components by solving Eq (4). Then, we invert the projection:

$$\begin{array}{ccc} X_{corruption} & = & L^{rpca}+S^{rpca}\;\;{\;}_{\text{(orginal}\;\text{RPCA)}}\\ & = & Y_{corruption}R^{-1}=\left(L_{Y}^{rpca}+S_{Y}^{rpca}\right)R^{-1}\\ & ≜ & {\bar{L}}^{rpca}+{\bar{S}}^{rpca}\;\;{\;}_{\text{(RP-RPCA)}} \end{array}$$

where we define ${\bar{\text{L}}}^{rpca}≜{\text{L}}_{Y}^{rpca}{\text{R}}^{-1}$ and ${\bar{\text{S}}}^{rpca}≜{\text{S}}_{Y}^{rpca}{\text{R}}^{-1}$.

**Fig 4:**
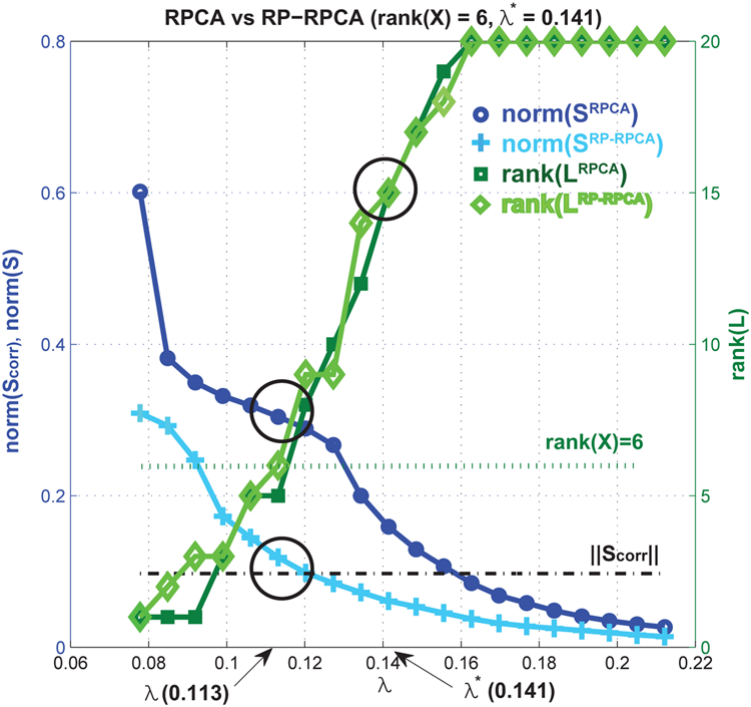
Statistics of a numerical example. We run RPCA for $X_{corruption}$ and $Y_{corruption}$ (we added sparse corruption to X). Left *y*-axis represents the norm of sparse component and the right *y*-axis shows the rank of **L** *(more detailed information in* S1 Fig. *and* S2 Fig.)

Fig 4 shows statistics of both RPCA and RP-RPCA (in which RPCA is applied to the matrix X and Y respectively) as a function of the tuning parameter *λ* in equation (4). In this example, $\lambda^{*}=1/\sqrt{\max(q,n\cdot N_{T})}=1/\sqrt{50}$. Since our input is still sparse in this example, the rank of both ${\text{L}}^{rpca},{\bar{\text{L}}}^{rpca}$ is 15 for λ* = 0.141 (rank(X)=6). If we choose *λ* = 0.113 (20% discounting the penalty for sparse component), the ranks of ${\text{L}}^{rpca},{\bar{\text{L}}}^{rpca}$ are approximately 6, which is the same as the rank of the original input X. With this choice of *λ*, for RPCA we find that ‖**S** ^*rpca*^‖ is much bigger than the original corruption signal $\left\|X_{corrpution}-X\|=\|S_{corruption}\right\|$. On the other hand, for RP-RPCA, we have $\left\|{\bar{\text{S}}}^{rpca}\|\approx \|{\text{S}}_{corruption}\right\|$. Therefore, for RP-RPCA, the separation of the low-rank component and sparse component is close to the true solution; for the original RPCA, there is mis-identification in both low-rank and sparse components due to the identifiability issue *(more detailed information is provided in* S2 Fig.: *we compare the original data element by element with the reconstruction result)*.

### 4.2 Application to Neural Data

Fig 3(left) shows the recorded neural activity aligned with submovement onset. The aligned neural activity shows that the ratios between units’ mean firing rates are fairly constant from the salient vertical striations in the plots and that temporal patterns exist across all the submovements. Also, as mentioned previously, the neural population activities are sparsely active (white color represents 0 spikes/sec) and show eccentric behavior; for example, some neurons have a much higher spiking rate than others.

Fig 3 shows the low-rank matrix from both RPCA (middle) and RP-RPCA (right) respectively (for simple comparison, we choose *m* = *n*). Since X is sparse and has an eccentric distribution, the singular vectors may not be reasonably spread out. Thus, applying RPCA directly to X results in the low-rank component being composed of only highly modulated neural activity in Fig 3(middle). On the other hand, RP-RPCA can extract the low-rank component from a more distributed set of neural dimensions than RPCA alone can. Therefore, the result of RP-RPCA gives a more visually appealing solution than the result of RPCA.

**Fig 5:**
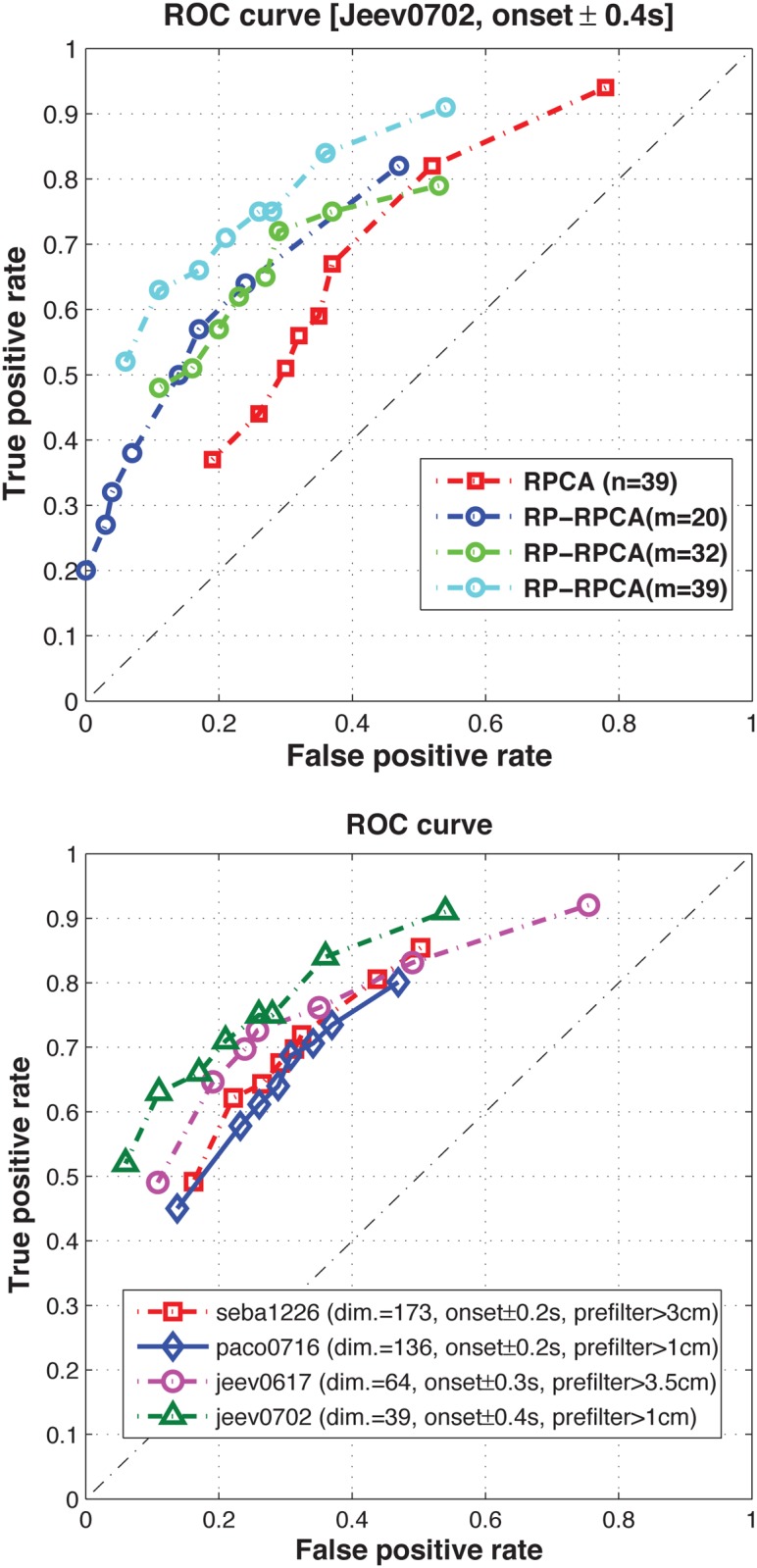
Receiver Operating Characteristic (ROC) curve of the prediction of submovement onset. (a) comparison between RPCA and RP-RPCA (target jumps task) (b) different monkeys or tasks where we prefiltered certain submovements with small amplitude in order to avoid artifacts of overfitting.

Since we extract neural features which represent common dynamic patterns across many experimental trials, we can use these features to detect and predict the onset of submovements. Here, we simply use the correlation between the extracted neural features from the training data set and the neural signals in the test data set [15]. For a practical purpose, we choose a correlation threshold and if the correlation is over the chosen threshold, we label a submovement onset as detected. In Fig 5, we vary thresholds for correlation score and show the receiver operating characteristic (ROC) curve of the prediction result. Since we consider different subjects and tasks, each curve shows the prediction performance for the corresponding subject and task respectively. To accurately predict submovement onset times found by submovement decomposition, the correlation function should peak around the movement onset time. The following observations suggest the potential application of RP-RPCA to predict movement execution in a closed-loop Brain Machine Interface (BMI) system:

- **(observation 1)** Fig 5(a) represents the ROC curve of the prediction of submovement onset time. Since RP-RPCA can handle the identifiability issue, we can see that the overall prediction performance based on RP-RPCA is better than the performance based on RPCA; we can reduce the false positive rate while increasing the true positive rate.
- **(observation 2)** Fig 5(b) shows the ROC curves of the prediction of submovement onset for different subjects or various tasks including center-out task and random-pursuit. This prediction could allow correction of movement execution errors in a closed-loop BMI system. Note that instead of applying the proposed method to only one subject [15], we apply it for different subjects including various tasks to generalize the use of our method.

In this section, we applied the proposed method to neural data which are naturally sparse and have eccentric distribution. We explored the benefits of using RP while preserving certain statistical characteristics of aggregate neural activity, and showed the improvement of the overall submovement prediction performance by identifying neural features properly.

### 4.3 Application to drug-induced perturbation experiments

In this section, we consider multidimensional spatio-temporal data sets from gene regulatory networks with various perturbation experiments. Since in the previous section, we evaluated the performance of the RP-RPCA method and demonstrate advantages over the RPCA method by properly handling identifiability issue caused by sparsity or eccentric distribution on the simulated and neural data, we directly apply the RP-RPCA method here and focus on explanations of some biological findings, which are consistent with biological knowledge from the references.

**Fig 6:**
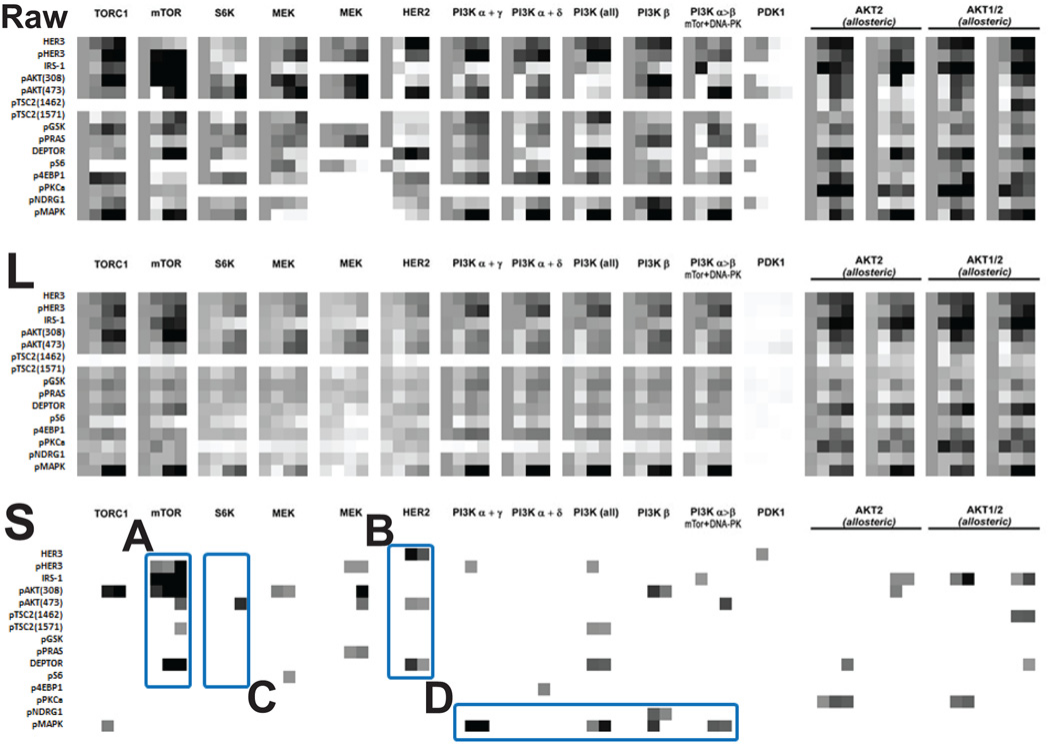
Drug-induced perturbation experiments [5] (16 perturbations×15 gene expressions×4 time points [0, 1, 48, 72h]): (upper) raw data (middle) low-rank component and (lower) highly aberrant sparse component using threshold, where **TORC1**, **mTOR**, **S6K**, **MEK**(1), **MEK**(2), **HER2**, **PI3K**(*α* + *γ*, *α* + *δ*, all, *β*, *α* > *β*), **PDK1**, **AKT2**(1), **AKT2**(2), **AKT1/2**(1) and **AKT1/2**(2) represents various perturbations and **HER3**, **pHER3**, **IRS**-1, **pAKT(308)**, **pAKT(473)**, **pTSC(1462)**, **pTSC2(1571)**, **pGSK**, **pPRAS**, **DEPTOR**, **pS6**, **p4EBP1**, **pPKCa**, **pNDRG1** and **pMAPK**are the measured expressions.

We consider drug-induced perturbation experiments using **SKBR3** cell line [5] which has been used in studies of Human Epidermal Growth Factor Receptor2 (HER2) positive breast cancer. We choose this data set because it has 16 perturbations using a single cell line and contains 15 gene expressions with 4 time points as shown in Fig 6(top row). The middle row represents the low-rank component **L** and the bottom row represents the highly aberrant sparse component **S**. In raw data (top row), nearly all treatments show differential responses and thus, visually comparing gene expressions and searching the featured responses may not be obvious tasks, especially without any *a priori* information about the underlying system. However, the result of the proposed method shows that the low-rank component (middle row) can be categorized into approximately 3-4 featured responses as shown in Fig 6(middle row), and the sparse component (bottom row) shows specific genomic aberration responses which are consistent with biological understanding where the details will be described below. Note that we do not use any prior knowledge about the underlying system to separate these data sets into the low-rank component and the sparse component. Also, since we solve the optimization problem (4), this decomposition is not subjective and it enables us to focus on the precise effects of each particular features by placing emphasis on the commonalities.

Also, the following observations suggest mechanisms of response and resistance which may inform unanticipated biological insight.

• (**Observation 1**, **A** **in** Fig 6) **mTOR** inhibition shows aberration responses in **DEPTOR**, **pHER3**, **IRS-1** and **pAKT(308, 473)** across other drug-induced perturbation results. However, it is unclear as how to distinguish these responses by visual inspection in the raw data matrix (i.e., Fig 6, top row) without any *a priori* information.
Also, in [22], **DEPTOR** is identified as an **mTOR**-interacting protein whose expression is negatively regulated by **mTORC1** and **mTORC2**. Also, Peterson *et al*. found that **DEPTOR** overexpression suppresses **S6K1** but it activates **AKT** by relieving feedback inhibition from **mTORC1** to **PI3K** signaling. Therefore, for **mTOR** inhibition, high **DEPTOR** expression is necessary to maintain **PI3K** and **AKT** activation as shown in Fig 6A which is consistent with the result [22].
• (**Observation 2**, **B** **in** Fig 6) **HER2** inhibition results in aberration responses of **HER3**, **pAKT(473)** and **DEPTOR**. S3 Fig. [23] represents an abstract model of **HER2** overexpressed breast cancer where **PHLPP** isoforms are a pair of protein phosphatases, **PHLPP1** and **PHLPP2**, which are important regulators of **AKT** serine-threonine kinases (**AKT1, AKT2, AKT3**) and conventional protein kinase C (**PKC**) isoforms. **PHLPP** may act as a tumor suppressor in several types of cancer due to its ability to block growth factor-induced signaling in cancer cells [24]. **PHLPP** dephosphorylates **SER473** (the hydrophobic motif) in **AKT**, thus partially inactivating the kinase [25].
High **DEPTOR** expression indicates low **mTORC1** and **mTORC2** [22], and according to the model in S3 Fig., the amounts of the activated **HER3** and **AKT** are increased by relieving inhibition reactions. The more interesting fact is that **PHLPP** is known to dephosphorylate **SER473** in **AKT** (i.e., partially inactivating the kinase) which is captured in the sparse component **pAKT(473)** in Fig 6B.
• (**Observation 3**, **C** **in** Fig 6) **S6K** inhibition results in aberration responses of **pAKT(473)**. Since **S6K** is located downstream of the **AKT-TSC2-mTORC** pathway and fed back to **pAKT(473)**, **S6K** inhibition captures only activation of **pAKT(473)**. Specifically, our result is consistent with the partial inactivating characteristics of **PHLPP** (i.e., **mTOR** → **PHLPP** ⊣ **pAKT(473)**)[25].
• (**Observation 4**, **D** **in** Fig 6) **PI3K** inhibition leads to increase more phosphorylation of **MAPK** compared to other perturbations.

We separate the common response from the heterogeneous responses using the proposed method without any prior information and the observations from the sparse components inform biological insights. We validate these insights compared with biological understanding from the references. One may argue that in some cases, we may draw these observations by the visual inspection of the raw data. However, since visual inspection is often subjective, we cannot convince ourselves, especially without any prior knowledge. In addition, as the dimension of high-throughput data increases, analysis based on visual inspection is not possible in practice. On the other hand, the proposed method helps us examine and analyze the large-scale features and then focus on the interesting details such as the **Observation 1-4** here. Since the proposed method does not use any prior information, it can provide us a more un-biased and objective way to interpret biological multi-dimensional data sets. Thus, we can also use the proposed method parallel to visual inspection with prior knowledge in order to validate our understanding based on the visual inspection more convincingly.

**Fig 7:**
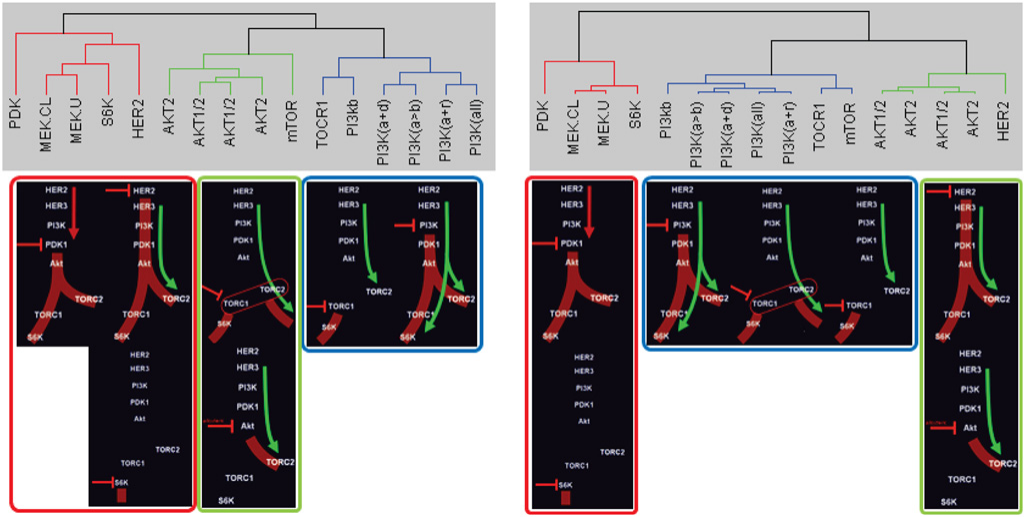
Clustered group. (left) hierarchical cluster and (right) the proposed method. Both clustered results compare with schematic overview of time series gene expression data set generated by M. Moasser.

Also, since abnormal behaviors or different responses to external stimuli or different cell lines can be extracted from the information available in the data set, we could cluster data correctly and reveal biological meaningful subtypes (**see Supplementary Information: Cluster Analysis** for details). Fig 7(top) shows the clustered result of these drug-induced perturbation experimental data set using existing hierarchical clustering (left figure, using raw data X with dissimilarity measure, *d*_*xy*_ in (S1) where the dissimilarity measure can effectively remove changes in the average measurement level or range of measurement from one sample to the next and it is widely used for biological applications) and the proposed method (right figure, using [**L S**] with *d*_*ϕψ*_ in (S2)) respectively. Also, Fig 7(bottom) represents schematic overview of time series gene expression data set as shown in Fig 6(top, raw data) with known graph structure. Thus, these diagrams summarize time series gene expressions such as the immediate effects of drug-induced perturbation that establish the new steady state and the compensatory responses. For example, negative perturbations (red dash bar) show the immediate effects on down regulation of signaling at the immediate target and other proteins (these are shown in red). The compensatory responses such as upregulation occur at later time points (these are shown in green). In order to compare the clustered result with each other, we arrange these schematic overviews with respect to our cluster results. We can easily see that our clustered result (right) is more consistent with the known gene regulatory network structure and responses than the result of existing hierarchical clustering (left). For example, hierarchical clustered result (left) shows that **HER2** and **mTOR** assigned to substantially different clusters.

### 4.4 Application to RPPA (Reverse Phase Protein Arrays) data set

**Fig 8:**
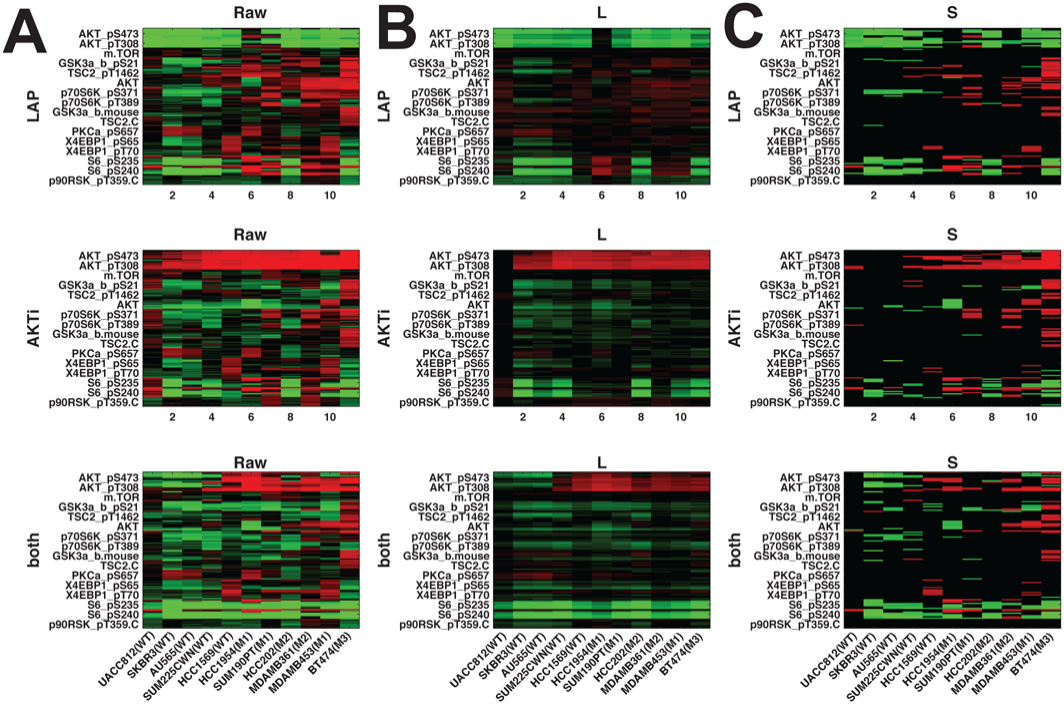
Application to RPPA data set. x-axis represents time steps ([0.5hr 1hr 2hr 4hr 8hr 72hr] for Raw data / Low-rank (L) / Sparse (S) respectively): (**A**) raw data 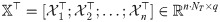 (**B**) low-rank component **L** and (**C**) highly aberrant sparse component **S** using threshold (**WT**: wild type, **M1**: H1047R (kinase domain mutation), **M2**: E545K (helical domain mutation), and **M3**: K111N mutation in **PIK3CA**).

Breast cancers are comprised of distinct subtypes which may respond differently to pathway-targeted therapies as shown in Fig 8A; collections of breast cancer cell lines show differential responses across cell lines and show subtype-, pathway-, and genomic aberration-specific responses [2]. Fig 8A shows the raw data $X^{⊤}=\left[\begin{array}{c} {𝒳}_{1}^{⊤};{𝒳}_{2}^{⊤};\ldots ;{𝒳}_{n}^{⊤} \end{array}\right]\in R^{n\cdot N_{T}\times q}$, Fig 8B represents the common response and Fig 8C represents the aberrant responses. These observations suggest mechanisms of response and resistance which differ across cell lines. Here, we use a data set generated in the Gray Lab using Reverse Phase Protein Arrays (RPPA) from the Mills Lab [26] which presents a time course analysis on 11 cell lines (all **HER2** amplified: 5 wild-type and 6 **PI3K** mutant cell lines) in response to **Lapatinib**, **AKT** inhibitor and combination of the two. The time course for RPPA is at 30min, 1h, 2h, 4h, 8h, 24h, 48h and 72h post-treatment.

**Fig 9:**
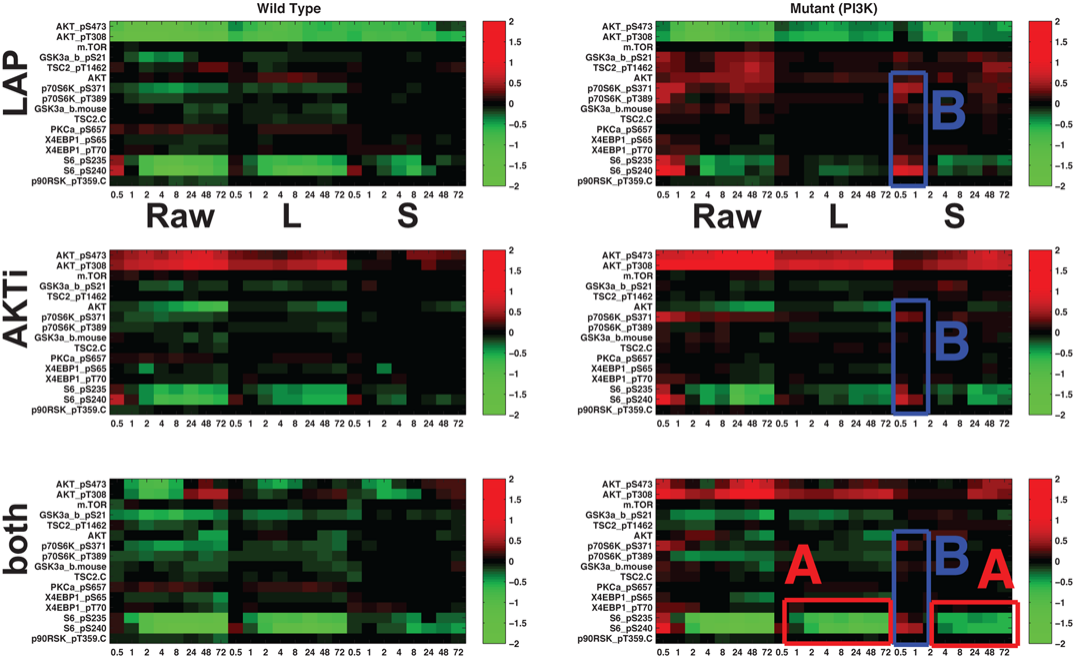
Heat maps showing average response based on both raw data and disentanglement result within subtype to targeted therapeutics in Fig 8: (left) **HER2+**/**wild type**, (right) **HER2+**/ **PI3K mutant**. Each representation consists of average responses of raw RPPA, low-rank component and sparse component. Each row represents targeted therapeutics alone and in combination (**LAP, AKTi, both**). (A) In the **PI3K** mutation with applying both inhibitors, full inhibition of **pS6RP** is observed (B) the main difference between wild-type and **PI3K** mutant is the response of **pS6RP** and **p70S6K**.

Since we are interested in analyzing different responses to external stimuli according to the cell line characteristics such as wild type- and **PI3K** mutant- cell lines, we average responses based on both raw data and disentanglement results shown in Fig 8 within subtype, and the averaged responses are shown in Fig 9. In Fig 9(top row), **Lapatinib** treatment(top row) results in immediate down-regulation of a variety of phosphoproteins in the signaling pathway. From the low-rank component (**L**), we can easily observe down-regulation and slow-recovery of the levels of activation, but the levels of activation are higher in the **PI3K** mutation cell lines (right). Treatment with **AKT** inhibitor(middle row) leads to immediate down-regulation of proteins (downstream of **AKT**) in all **HER2** amplified cell lines, although the amplitude of down-regulation is slightly less in cell lines with **PI3K** mutations. In the **PI3K** mutation cell lines, treatment with the combination of **Lapatinib** and **AKT** inhibitor leads to further down-regulation of the **AKT** signaling pathway but **AKT** levels are intermediate in comparison to those observed with inhibitor alone. Although these observations are still interesting, more interesting details might be in both the low-rank component **L** and the sparse component **S**:

- (**Observation 1 in** Fig 8) **BT474** shows highly aberrant behavior as shown in Fig 8. The mutation in **PIK3CA** has not been reported in any other samples and confers weak oncogenicity, unlike the typical hotspot **PIK3CA** mutations in the helical and kinase domains [27].
- (**Observation 2**, **A** **in** Fig 9) In the **PI3K** mutation with applying both inhibitors, full inhibition of **pS6RP** is observed in Fig 9 (in the sparse component) and these results show the synergistic effect of **Lapatinib** and **AKT** inhibitor (in the bottom row, low-rank component).
- (**Observation 3**, **B** **in** Fig 9) The main difference between wild-type and **PI3K** mutant is the response of **pS6RP** and **p70S6K**. For the wild-type cell lines, all treatments result in down-regulated **pS6RP** and **p70S6K**. However, for **PI3K** mutant cells, all treatments result in up-regulation **pS6RP** and **p70S6K** in the short-term (red in Fig 9B) and down-regulation in the long-term. Suppressing **pS6RP** relieves feedback inhibition and activates **AKT**. This difference makes **PI3K** mutation cells more resistant to **HER2** inhibitors than their wild-type counterparts. This finding is not obvious when we take a look at the raw data, especially Fig 8; it is really hard to differentiate common dynamic behavior from aberrant responses by visual inspection across cell lines. Thus, our method makes our finding more convincing not by visually searching X, but by finding these effect automatically by separating common response (**L**) and aberrant behavior (**S**) by solving (4).

## Discussion

Clustering and network inference are usually developed independently. For instance, until recently, most studies of gene regulatory network inference focus on a particular data set to identify the underlying graph structure, and apply the same method to other data sets and so on. Or, clustering methods are used on various data sets to find subgroups or classify them. However, we would argue that there are deep relationships between clustering and network inference and they can potentially cover each other’s shortcomings. For example, recent studies [28] [29] exploit the relationship between clustering and network inference and infer regulatory programs for individual genes to reveal module-level organization of regulatory networks. Since spatio-temporal gene expression patterns result from both the network structure and the integration of regulatory signals through the network [30], we might reveal the subtype graph structure and understand heterogeneity across various perturbations by comparing gene expression levels in the various perturbation conditions.

In this paper, we demonstrate that the proposed method helps to find distinct subtypes and classify dynamic responses in a robust way. In order to interpret multi-dimensional spatio-temporal data sets, it is common to compare the responses over experiments and find differences by looking at the raw data with prior knowledge. As the dimension of high-throughput data increases, interpreting large scale data sets is infeasible by inspection alone. For instance, we might have to consider multi-dimensions such as positive perturbation, negative perturbation, temporal response, various read-outs, mechanisms and various doses together. The proposed method provides a way to interpret multi-dimensional data sets. The low-rank representation provides the large-scale features and the sparse component shows the interesting details such as genomic aberration-specific responses. The intuition behind this is that one can recover the principal components of a data matrix even though a positive fraction of its entries are arbitrarily corrupted or a fraction of the entries are missing as well [13]. Thus, the notion of common dynamic feature is important for our analysis. We note this goes beyond the results in [14], i.e., steady-state analyses. In [14], since they consider steady-state analysis (no dynamic model), the sparse components only reflect the outliers or corruptions. However, we can identify drug-specific responses by extracting common temporal responses across various perturbation experiments. Hence, if there exists no common dynamic response, we may fail to disentangle the input data into low-rank and sparse components. Also, similar to video surveillance application in which the RPCA discriminates the motionless object as a low rank component, if drug-induced perturbations only affect a few genes, the common dynamic feature may be biased, i.e., dynamics of the unperturbed genes may be discriminated as a low rank component which may cause bias in analysis. Therefore, we should perturb our system uniformly well in order to extract the common dynamic feature correctly, and this is corresponding to the assumption for identifiability [13], i.e., sparse component is selected uniformly at random.

Also, although there is a wealth of literature describing canonical cell signaling networks, little is known about exactly how these networks operate in different cancer cells or different drug-induced perturbations. Our method can reveal aberrant responses or drug-specific responses across various stimuli or different cell lines by isolating the common dynamic responses from the raw data. Furthermore, a possible extension of the proposed method is that once we extract common responses, we apply inference algorithms to identify the unified structure using these common responses. Or, we can also focus on individual sparse components to identify the heterogeneity of network structure across cells of different types. Advancing our understanding of how these networks are deregulated across cancer cells and different targeted therapies will ultimately lead to improve effectiveness of pathway-targeted therapies.

Moreover, for a gene regulatory network application, since the number of time points is limited with respect to the number of proteins, we chose reasonable size proteomic data. Note that the proposed method use common dynamic features and thus we need a reasonable number of time steps. However, many proteome-wide or genome-wide data only include one time point (steady-state) or only a few time steps. Therefore, applying this method to large-scale real datasets with many time points is our current and future research and to this goal, we are currently collaborating with the groups which generate proteome-wide data with more time points.

## Conclusion

In this study, we develop a new method for clustering and analyzing multi-dimensional biological data. We illustrate how the proposed method can be useful to extract common event-related neural features across many experimental trials. Also, with time series gene expression data set, we show that the proposed method helps to find distinct subtypes and classify data sets in a robust way by separating common response and abnormal responses without any prior knowledge.

## Ethics Statement

(Experiments involving a non-human primate) All procedures were conducted in compliance with the National Institute of Health Guide for Care and Use of Laboratory Animals and were approved by the University of California, Berkeley Institutional Animal Care and Use Committee.

## Supporting Information

S1 Cluster Analysis (PDF)

S1 Fig (a) (upper) Input matrix X and singular value decomposition (SVD) ($X={\text{U}}_{x}{\text{Σ}}_{x}{\text{V}}_{x}^{*}$). (lower) Randomly projected input matrix Y and SVD ($Y={\text{U}}_{y}{\text{Σ}}_{y}{\text{V}}_{y}^{*}$). Note that since rank(X)=6, $U_{x}\in R^{q\times 6}$, $\Sigma_{x}\in R^{6\times 6}$, $V_{x}^{*}\in R^{6\times n\cdot N_{T}}$. In order to show how well singular vectors are spread out, we show the absolute value of each component. White represents zero value. (b) RPCA results. We run RPCA for sparsely corrupted $X_{corruption}$, $Y_{corruption}$. (we added sparse corruption to X as shown in S2 Fig.) Left *y*-axis represents the norm of X−L and the right *y*-axis shows the rank of **L**.
(TIF)

S2 Fig The out of RPCA and RP-RPCA with two different *λ* values. (a) For *λ* = 0.113, both **L** ^rpca^ and **L** ^rp-rpca^ have rank 6 (≈ rank(X)) as shown in Fig 4(b). There is a big difference between **S** ^rpca^ and the constructed corrupted signal ($X-X_{corr}$) (b) For λ* = 0.141, **S** ^rp-rpca^ is close to $X-X_{corr}$ but the low-rank components are misidentified by both RPCA and RP-RPCA because both **L** ^rpca^ and **L** ^rp-rpca^ have rank 15. Therefore, for RP-RPCA, the separation of the low-rank component and sparse component is close to the true solution but for original RPCA, we have misidentification in both the low-rank and sparse components. We can easily see that **S** ^*rpca*^ shows characteristics of the low-rank component in S2 Fig. (middle columns of each panel).
(TIF)

S3 Fig Abstract HER2 overexpressed breast cancer model. Red arrow represents activation and blue dash bar represents inhibition.
(TIF)

S4 Fig Simple cluster analysis. (a) green solid line with circle represents *y*_*corr*_(= *y*_*L*_ + 0) and blue solid line with circle represents *x*_*corr*_(= *x*_*L*_ + *x*_*S*_) where filled circle represents corrupted data, unfilled circle represents uncorrupted data (*x*_*L*_) and unfilled square represents corruption signal (*x*_*S*_) (b) *x*_*corr*_-*y*_*corr*_ plot with 1-*correlation* distance (*d*_*xy*_) without modification(left), with disentanglement(middle), and with disentanglement/weighting factor *γ*.
(TIF)
