# Supplementary material for: Disentangling Multidimensional Spatio-Temporal Data into Their Common and Aberrant Responses
Source: PLoS One. 2015 Apr 22;10(4):e0121607. doi: 10.1371/journal.pone.0121607 (PMC4406848; doi:10.1371/journal.pone.0121607)
Supplement: S1 Cluster Analysis — (PDF) [file pone.0121607.s001.pdf]

Supporting Information

S1. Cluster Analysis

Overview: Dissimilarity

Common measures of dissimilarity for data include Euclidean distance [12], $\|x-y\| = \sqrt{\sum_{i=1}^{p}(x_i - y_i)^2}$ where x and y are p-vectors of measurements on the objects to be clustered. Also, Manhattan distance $d_{xy} = \sum_{i=1}^{p}|x_i - y_i|$ is used, and the "1-*correlation*" distance is defined as follows

$$d_{xy} = 1 - \rho_{xy} = 1 - \frac{\sum_{i=1}^{p}(x_i - \bar{x})(y_i - \bar{y})}{[\sum_{i=1}^{p}(x_i - \bar{x})^2]^{1/2}[\sum_{i=1}^{p}(y_i - \bar{y})^2]^{1/2}} \quad \text{(S1)}$$

The 1-*correlation* distance is bounded in $[0, 2]$. This dissimilarity is invariant to changes in location or scale of either x or y. The 1-*correlation* dissimilarity can be related to the more familiar Euclidean distance: if $\tilde{x} = \frac{x-\bar{x}}{\sqrt{\sum_i (x_i-\bar{x})^2/p}}$ and $\tilde{y} = \frac{y-\bar{y}}{\sqrt{\sum_i (y_i-\bar{y})^2/p}}$, then $\|\tilde{x} - \tilde{y}\|^2 = 2p(1-\rho_{xy})$. That is, squared Euclidean distance for standardized objects is proportional to the correlation of the original objects. For microarray data, the choice of a dissimilarity measure makes it a popular choice for biological applications. Changes in the average measurement level or range of measurement from one sample to the next are effectively removed by this dissimilarity.

Missing data and corruption

Suppose we have large corruptions or outliers in data. If we use existing clustering method based on Euclidean distance, these corruptions or outliers can distort measure of dissimilarity and thus may lead to improper clustering. We propose the method to handle these outliers before calculating the correlation and thus, we can provide more appealing clusters.

For example, we consider highly-correlated signal $x_L = \sin(t) + n_1$ and $y_L = \sin(t) + n_2$ where t is time step and n_1, n_2 are Gaussian noise $\mathcal{N}(0, \sigma)$. Now, we add a sparse corruption (x_S) to the original signal (x_L) as shown in S4 Fig. (a) and calculate the dissimilarity between $x_{corr}(= x_L + x_S)$ and $y_{corr}(= y_L + 0)$. Even though we choose the d-sparse corruption of x_S where $d(\ll p)$ is the number of nonzero component in x_S, the correlation is degraded as shown in S4 Fig. (b)(left). Assuming that we know the corruption signal x_S and y_S, we can decompose x_{corr}, y_{corr} as $\phi = [x_L; x_S] \in \mathbb{R}^{2p}$ and $\psi = [y_L; y_S] \in \mathbb{R}^{2p}$ respectively. In S4 Fig. (b)(middle), the red square represents the corruption signal where $y_S = 0$. Since corruption signal changes the mean and the variance, the correlation is still degraded in (b)(middle). We introduce γ as a turning parameter so that we allow different weighting factors for (x_L, y_L) and (x_S, y_S) respectively. For example, we choose small γ for the corruption signal (x_S, y_S).

Therefore, in order to deal with corrupted signals or abnormal responses and cluster them, we should separate the original signal from these corrupted or abnormal signals first and then calculate the dissimilarity with adjusting weighting factor γ. Without considering dynamics (static data), these corrupted data may simply represent outliers. However, when we include dynamics, they may represent meaningful or condition-specific responses.

Our approach: a new *1-correlation* distance

We rewrite the "1-*correlation*" distance (S1) as $d_{xy} = 1 - \frac{\tilde{x} \cdot \tilde{y}}{\|\tilde{x}\|\,\|\tilde{y}\|}$ where $x, y \in \mathbb{R}^p$, $\tilde{x} \triangleq x - \bar{x} \cdot \mathbf{1}_p$, $\tilde{y} \triangleq y - \bar{y} \cdot \mathbf{1}_p$ and $\mathbf{1}_p = \underbrace{[1 \quad \ldots \quad 1]}_{p}$ and consider the separation as follows: $\phi = \begin{bmatrix} x_{\mathbf{L}} \\ x_{\mathbf{S}} \end{bmatrix} \in \mathbb{R}^{2p}$ and $\psi =$

$\begin{bmatrix} y_{\mathbf{L}} \\ y_{\mathbf{S}} \end{bmatrix} \in \mathbb{R}^{2p}$ where $x = x_{\mathbf{L}} + x_{\mathbf{S}}$, $y = y_{\mathbf{L}} + y_{\mathbf{S}}$ and the subscript $\mathbf{L}$,$\mathbf{S}$ represent low-rank component and sparse component. We define "1-*correlation*" distance for ϕ, ψ as follows:

$$d_{\phi\psi} = 1 - \rho_{\phi\psi} = 1 - \frac{\sum_{i=1}^{2p}(\phi_i - \bar{\phi})(\psi_i - \bar{\psi})}{[\sum_{i=1}^{2p}(\phi_i - \bar{\phi})^2]^{1/2}[\sum_{i=1}^{2p}(\psi_i - \bar{\psi})^2]^{1/2}} \tag{S2}$$

where $\bar{\phi} = \frac{1}{2p}\sum_{i=1}^{2p}\phi_i = \frac{1}{2}\bar{x}$ and $\bar{\psi} = \frac{1}{2p}\sum_{i=1}^{2p}\psi_i = \frac{1}{2}\bar{y}$. The relation between $d_{xy}(= 1 - \rho_{xy})$ and $d_{\phi\psi}(= 1 - \rho_{\phi\psi})$ is as follows:

$$d_{xy} = 1 - \frac{\hat{\phi} \cdot \hat{\psi}}{\left\|\hat{\phi}\right\| \left\|\hat{\psi}\right\|} \text{ and } d_{\phi\psi} = 1 - \frac{\tilde{\phi} \cdot \tilde{\psi}}{\left\|\tilde{\phi}\right\| \left\|\tilde{\psi}\right\|}$$

where $\tilde{x} \triangleq x - \bar{x} \cdot \mathbf{1}_p = \begin{bmatrix}\mathbf{I}_p & \mathbf{I}_p\end{bmatrix} \begin{bmatrix} x_L - \frac{\bar{x}}{2} \cdot \mathbf{1}_p \\ x_S - \frac{\bar{x}}{2} \cdot \mathbf{1}_p \end{bmatrix} = \begin{bmatrix}\mathbf{I}_p & \mathbf{I}_p\end{bmatrix} (\phi - \bar{\phi} \cdot \mathbf{1}_{2p}) \triangleq \begin{bmatrix}\mathbf{I}_p & \mathbf{I}_p\end{bmatrix} \tilde{\phi}$, $\tilde{y} \triangleq \begin{bmatrix}\mathbf{I}_p & \mathbf{I}_p\end{bmatrix} \tilde{\psi}$, $\mathbf{I}_p$ is p-dimensional identity matrix, $\hat{\phi} = \frac{1}{\sqrt{2}} \begin{bmatrix}\mathbf{I}_p & \mathbf{I}_p \\ \mathbf{I}_p & \mathbf{I}_p\end{bmatrix} \tilde{\phi} \triangleq \mathcal{P}_{xy}\tilde{\phi}$, $\hat{\psi} = \mathcal{P}_{xy}\tilde{\psi}$, $\mathcal{P}_{xy}^{\top}\mathcal{P}_{xy} = \begin{bmatrix}\mathbf{I}_p & \mathbf{I}_p \\ \mathbf{I}_p & \mathbf{I}_p\end{bmatrix} = \sqrt{2}\mathcal{P}_{xy} \succeq 0$ and $\mathcal{P}_{\phi\psi}^{\top}\mathcal{P}_{\phi\psi} = 1 \cdot \mathcal{P}_{\phi\psi} = \begin{bmatrix}\mathbf{I}_p & \mathbf{0}_p \\ \mathbf{0}_p & \mathbf{I}_p\end{bmatrix} \succ 0$.

Therefore, d_{xy} uses the mixture of low-rank component and sparse component but $d_{\phi\psi}$ calculates the correlation based on the separation. Also, in order to adjust the weighting factor as shown in S4 Fig. (b)(right), we simply denote $\mathcal{P}_{\phi\psi} = \begin{bmatrix}\mathbf{I}_p & \mathbf{0}_p \\ \mathbf{0}_p & \gamma\mathbf{I}_p\end{bmatrix}$ where γ is a weighting factor.

In fact, when the sparse component is zero, i.e., there is no corruption or outliers in the input data, $x_L = x$ and $y_L = y$. Then, the proposed method is identical to use 1-*correlation*, i.e., $d_{xy} = d_{\phi\psi}$. In other words, since we propose a modification of existing clustering method by separating the input data into the low-rank component and the sparse component, the proposed method is more general than 1-*correlation* metric.

Thus, even though a data set is partially corrupted or includes aberrant responses across different perturbations or cell lines (i.e., sparse component is nonzero), the proposed method ($d_{\phi\psi}$) still be able to calculate correct measures of dissimilarity by adjusting meaningful or condition-specific responses as shown in S4 Fig. (b). Also, in the missing value problem, since the sparse component represents the corruption which is not important, we can use only the low-rank component for clustering.
