# Supplementary figures and images for: Disentangling Multidimensional Spatio-Temporal Data into Their Common and Aberrant Responses
Source: PLoS One. 2015 Apr 22;10(4):e0121607. doi: 10.1371/journal.pone.0121607 (PMC4406848; doi:10.1371/journal.pone.0121607)

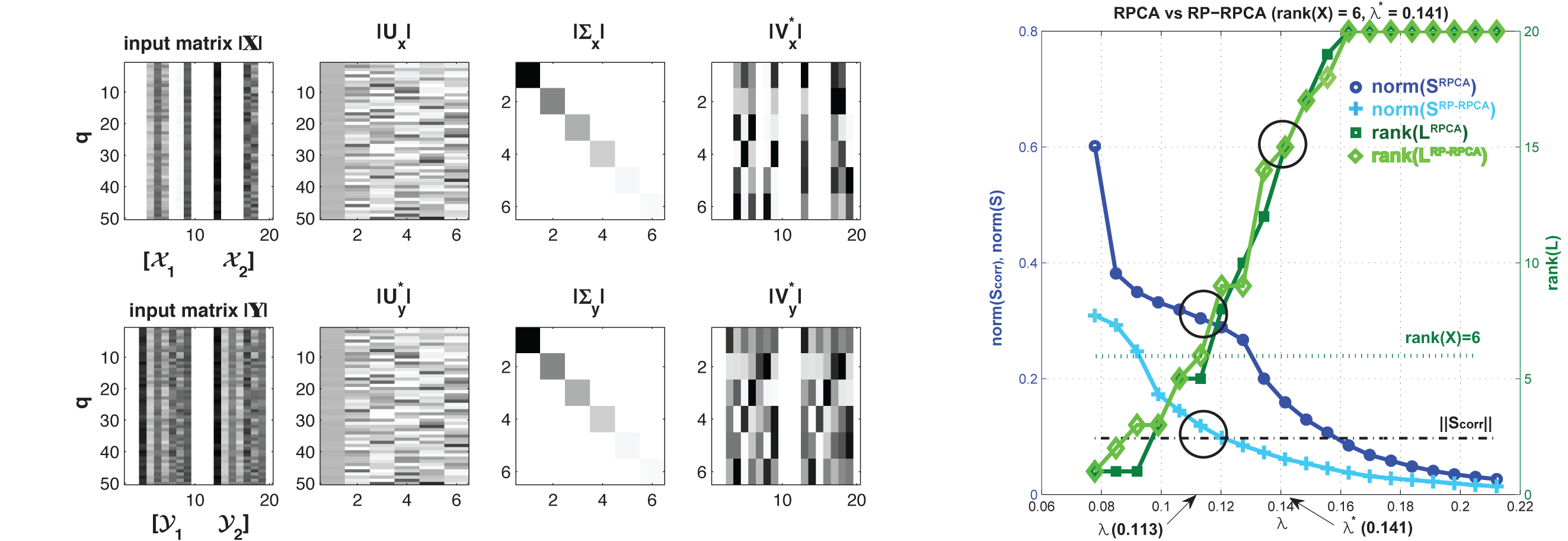

Supplement: S1 Fig — (lower) Randomly projected input matrix Y and SVD (Y=UyΣyVy*). Note that since rank(X)=6, Ux∈Rq×6, Σx∈R6×6, Vx*∈R6×n⋅NT. In order to show how well singular vectors are spread out, we show the absolute value of each component. White represents zero value. (b) RPCA results. We run RPCA for sparsely corrupted Xcorruption, Ycorruption. (we added sparse corruption to X as shown in S2 Fig.) Left y-axis represents the norm of X−L and the right y-axis shows the rank of L. (TIF) [file pone.0121607.s002.tif]

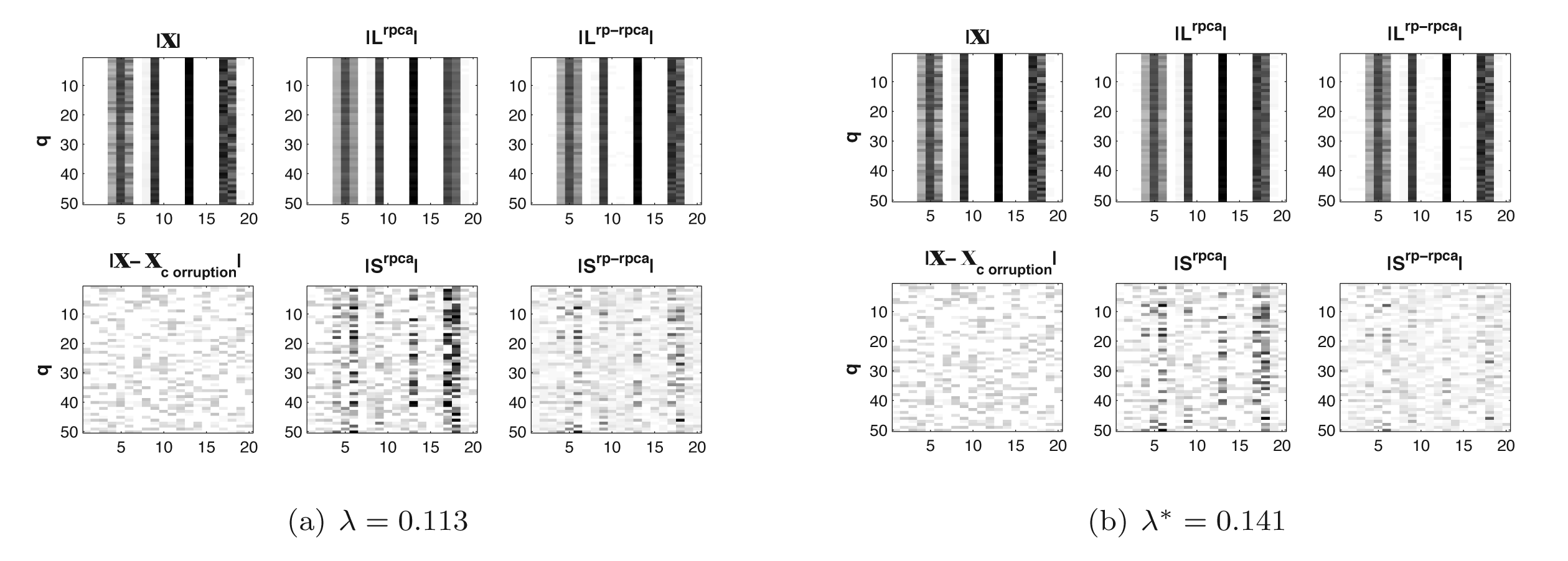

Supplement: S2 Fig — (a) For λ = 0.113, both L rpca and L rp-rpca have rank 6 (≈ rank(X)) as shown in Fig 4(b). There is a big difference between S rpca and the constructed corrupted signal (X−Xcorr) (b) For λ* = 0.141, S rp-rpca is close to X−Xcorr but the low-rank components are misidentified by both RPCA and RP-RPCA because both L rpca and L rp-rpca have rank 15. Therefore, for RP-RPCA, the separation of the low-rank component and sparse component is close to the true solution but for original RPCA, we have misidentification in both the low-rank and sparse components. We can easily see that S rpca shows characteristics of the low-rank component in S2 Fig. (middle columns of each panel). (TIF) [file pone.0121607.s003.tif]

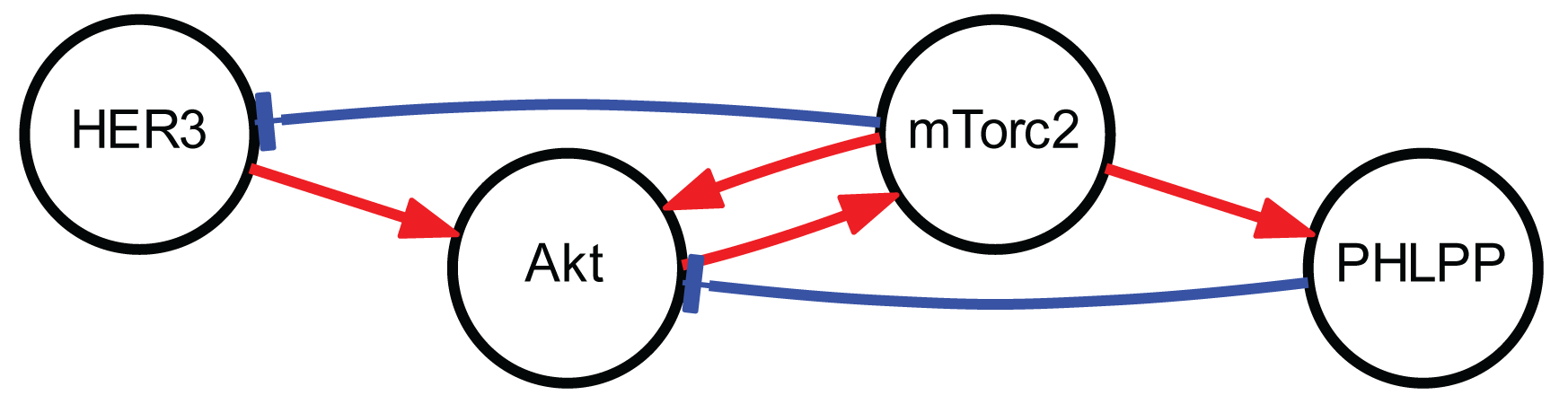

Supplement: S3 Fig — Red arrow represents activation and blue dash bar represents inhibition. (TIF) [file pone.0121607.s004.tif]

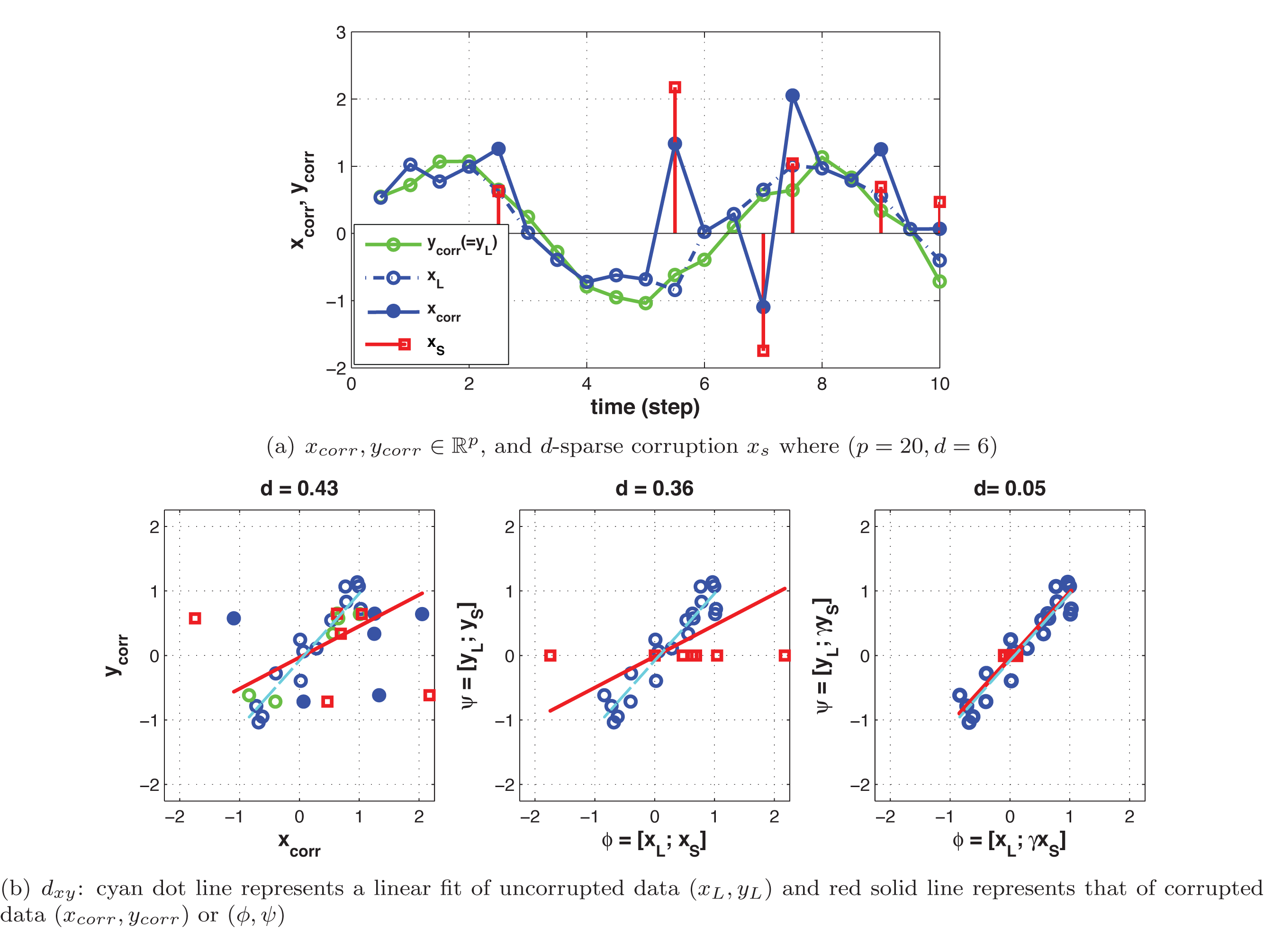

Supplement: S4 Fig — (a) green solid line with circle represents y corr(= y L + 0) and blue solid line with circle represents x corr(= x L + x S) where filled circle represents corrupted data, unfilled circle represents uncorrupted data (x L) and unfilled square represents corruption signal (x S) (b) x corr-y corr plot with 1-correlation distance (d xy) without modification(left), with disentanglement(middle), and with disentanglement/weighting factor γ. (TIF) [file pone.0121607.s005.tif]
